# Targeted Exome Sequencing Identified Novel *USH2A* Mutations in Usher Syndrome Families

**DOI:** 10.1371/journal.pone.0063832

**Published:** 2013-05-30

**Authors:** Xiu-Feng Huang, Ping Xiang, Jie Chen, Dong-Jun Xing, Na Huang, Qingjie Min, Feng Gu, Yi Tong, Chi-Pui Pang, Jia Qu, Zi-Bing Jin

**Affiliations:** 1 Division of Ophthalmic Genetics, Laboratory for Stem Cell & Retinal Regeneration, The Eye Hospital of Wenzhou Medical College, Wenzhou, China; 2 The State Key Laboratory Cultivation Base and Key Laboratory of Vision Science, Ministry of Health P. R. China, Wenzhou, China; 3 Center for Excellence in Low Vision and Visual Rehabilitation, The Eye Hospital of Wenzhou Medical College, Wenzhou, China; 4 The Institute of Genomic Medicine, Wenzhou Medical College, Wenzhou, China; 5 Fuzhou Southeastern Eye Hospital, Fuzhou, China; 6 Department of Ophthalmology and Visual Sciences, The Chinese University of Hong Kong, Hong Kong, China; Innsbruck Medical University, Austria

## Abstract

Usher syndrome (USH) is a leading cause of deaf-blindness in autosomal recessive trait. Phenotypic and genetic heterogeneities in USH make molecular diagnosis much difficult. This is a pilot study aiming to develop an approach based on next-generation sequencing to determine the genetic defects in patients with USH or allied diseases precisely and effectively. Eight affected patients and twelve unaffected relatives from five unrelated Chinese USH families, including 2 pseudo-dominant ones, were recruited. A total of 144 known genes of inherited retinal diseases were selected for deep exome resequencing. Through systematic data analysis using established bioinformatics pipeline and segregation analysis, a number of genetic variants were released. Eleven mutations, eight of them were novel, in the *USH2A* gene were identified. Biparental mutations in *USH2A* were revealed in 2 families with pseudo-dominant inheritance. A proband was found to have triple mutations, two of them were supposed to locate in the same chromosome. In conclusion, this study revealed the genetic defects in the *USH2A* gene and demonstrated the robustness of targeted exome sequencing to precisely and rapidly determine genetic defects. The methodology provides a reliable strategy for routine gene diagnosis of USH.

## Introduction

Usher syndrome (USH) is an autosomal recessive disorder characterized by visual loss due to retinitis pigmentosa (RP), sensorineural hearing impairment and variable vestibular dysfunction, with remarkable clinical and genetic heterogeneity [1]. According to the disease severity and progression, three subtypes of USH have been descrobed. Type I (USH1) is the most severe form characterized by prepubertal onset of RP, profound hearing loss, and vestibular dysfunction. Type II (USH2) is characterized by postpuberal onset RP and moderate deafness without vestibular dysfunction. Type III (USH3) is featured as postlingual deafness, teenage-onset RP and varying degree of vestibular dysfunction. To date, at least 12 loci have been mapped for the three types of USH in human chromosomes and 10 genes have been identified [2]. These genes totally comprise 321 coding exons spanning a length of 59,430-nt (Table S1). Reported mutations of USH widely spread over the coding regions of these causative genes. Genetic screening through traditional approaches, such as direct sequencing is therefore difficult. A high-throughout and cost-effective method to detect the genetic defects is needed. Targeted or whole exome sequencing has been proved to be a powerful tool to discover novel disease-related genes or genetic mutations in large genomic regions [3]. With the progresses on next-generation sequencing (NGS) and bioinformatics, it has been demonstrated to have higher efficiency but lower cost comparing with previous methods [4]. In this study, we utilized targeted exome sequencing (TES) to study genetic defects in five USH families and attempted to establish a strategy useful for genetic diagnosis of USH patients.

## Materials and Methods

### Patient Recruitment

**Figure 1:**
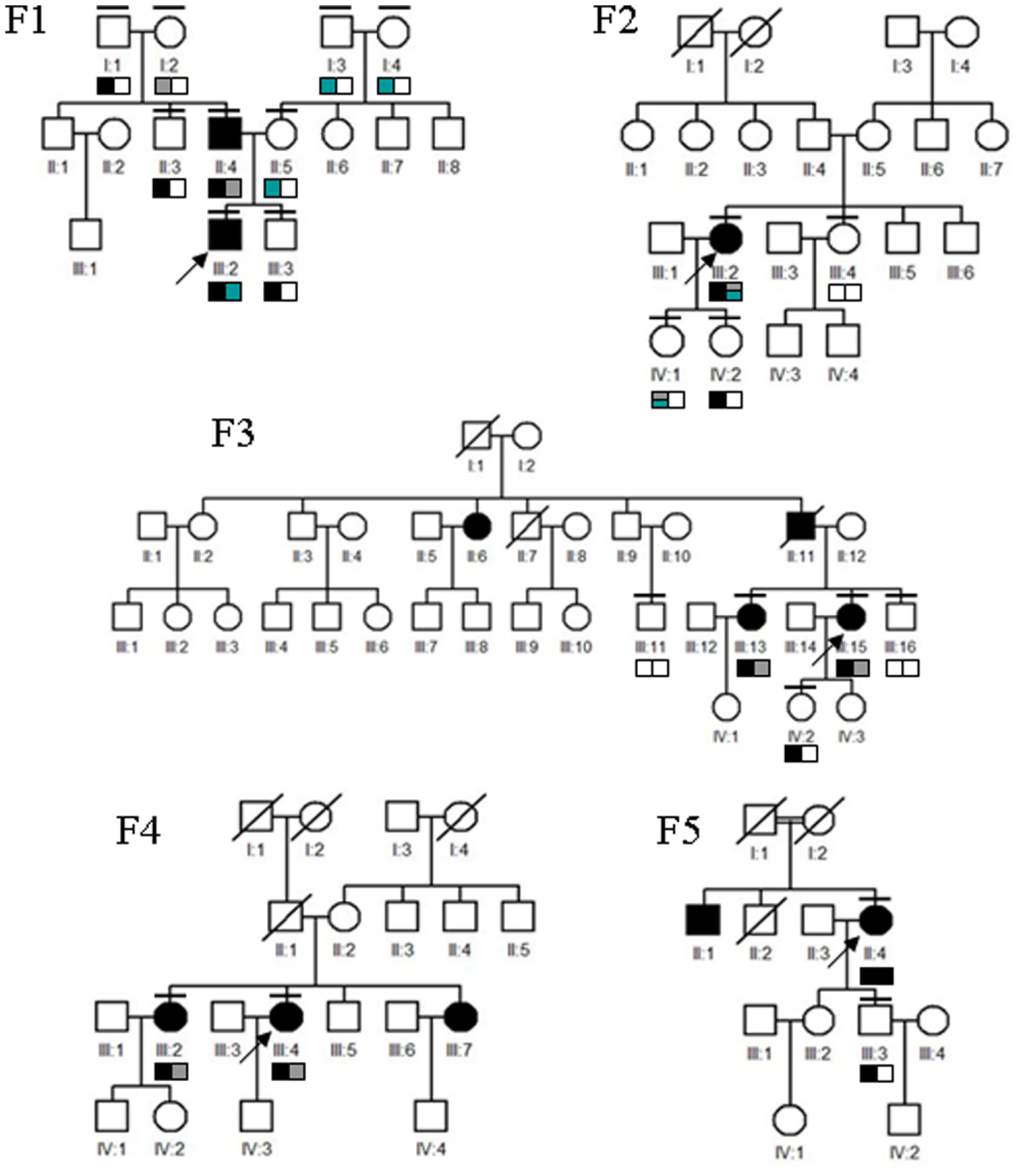
Families with Usher syndrome. Pedigrees of the Usher syndrome in this study. Closed symbol represents affected patient and open symbol indicates unaffected subject. The bar over the symbol indicates examined subjects in this study. Arrow indicates proband. Slash represents deceased person. The box labeled with different color indicates different mutation in each pedigree.

This study adhered to the tenets of the Declaration of Helsinki. The protocol was approved by the ethics committee of The Eye Hospital of Wenzhou Medical College and written informed consent has been obtained from all study subjects. Comprehensive ophthalmic examination, including routine eye tests, perimetry, electroretinography (ERG), and optical coherence tomography (OCT), were carried out in each patient. Detailed family history was obtained through personal interviews with patients and their relatives (Figure 1). Peripheral blood samples were collected from both the affected patients and unaffected kinships.

### Illumina Library Preparation

Genomic DNA was extracted from whole blood using a DNA Extraction kit (TIANGEN, Beijing) using the manufacturer's instructions. The DNA was quantified with Nanodrop 2000 (Thermal Fisher Scientific, DE). A minimum of 3 μg DNA was used for the indexed Illumina libraries according to manufacturer's protocol. The final library size 350 bp–450 bp including adapter sequences was selected.

### Disease Genes Enrichment and Sequencing

A total of 144 disease genes (Table S2) were selected by a gene capture strategy, using GenCap custom enrichment kit (MyGenostics, Beijing) based on previously described technologies [5], [6]. The biotinylated single-strand capture probes were designed to tile along the exonic non-repeated regions of the genes (Table S3). The capture experiment was conducted according to manufacturer's protocol. In brief, 1 μg DNA library was mixed with Buffer BL and GenCap probe (MyGenostics, Beijing), heated at 95°C for 7 min and 65°C for 2 min on a PCR machine; 23 μl of the 65°C prewarmed Buffer HY (MyGenostics, Beijing) was then added to the mix, and the mixture was hold at 65°C with PCR lid heat on for 22 hours for hybridization. 50 μl MyOne beads (Life Technology) was washed in 500 μL 1Xbinding buffer for 3 times and resuspended in 80 μl 1Xbinding buffer. Sixty-four μl 2Xbinding buffer was added to the hybrid mix, and transferred to the tube with 80 μl MyOne beads. The mix was rotated for 1 hour on a rotator. The beads were then washed with WB1 buffer at room temperature for 15 minutes once and WB3 buffer at 65°C for 15 minutes three times. The bound DNA was then eluted with Buffer Elute. The eluted DNA was finally amplified for 15 cycles using the following program: 98°C for 30 s (1 cycle); 98°C for 25 s, 65°C for 30 s, 72°C for 30 s (15 cycles) and 72°C for 5 min (1 cycle). The PCR product was purified using SPRI beads (Beckman Coulter) according to manufacturer's protocol. The enrichment libraries were sequenced on Illumina Solexa HiSeq 2000 sequencer for paired read 100bp.

### Bioinformatics Analysis

After Solexa HiSeq 2000 sequencing, high-quality reads were retrieved from raw reads by filtering out the low quality reads and adaptor sequences using the Solexa QA package [7] and the cutadapt program (http://code.google.com/p/cutadapt/), respectively. SOAPaligner program [8] was then used to align the clean reads to the reference human genome (hg19). After the PCR duplicates were removed by the Picard software, [9] the SNPs was firstly identified using the SOAPsnp program, [8] Subsequently, we realigned the reads to the reference genome using BWA [10] and identified the insertions or deletions (InDels) using the GATK program [11], The identified SNPs and InDels were annotated using the Exome-assistant program (http://122.228.158.106/exomeassistant). MagicViewer [12] was used to view the short read alignment and validate the candidate SNPs and InDels. Nonsynonymous variants were evaluated by four algorithms, PolyPhen, SIFT, PANTHER and Pmut as described previously, to determine pathogenicity [13]. Sequencing data were deposited in NIH Short Read Archive (PRJNA189497).

### Expanded Validation

DNA samples of all the family members (Figure 1) were taken for the same targeted exome sequencing and filtering strategy. Furthermore, coding regions of the mutations identified as described above were amplified by polymerase chain reaction (PCR) for conventional direct sequencing. Purified PSR products were cycle-sequenced on an ABI 3500 Genetic Analyzer (Applied Biosystems, CA). Sanger sequencing results were analyzed by Mutation Surveyor (Softgenetics, PA) and reconfirmed by the same procedure.

## Results

### Phenotypic Determination

**Figure 2:**
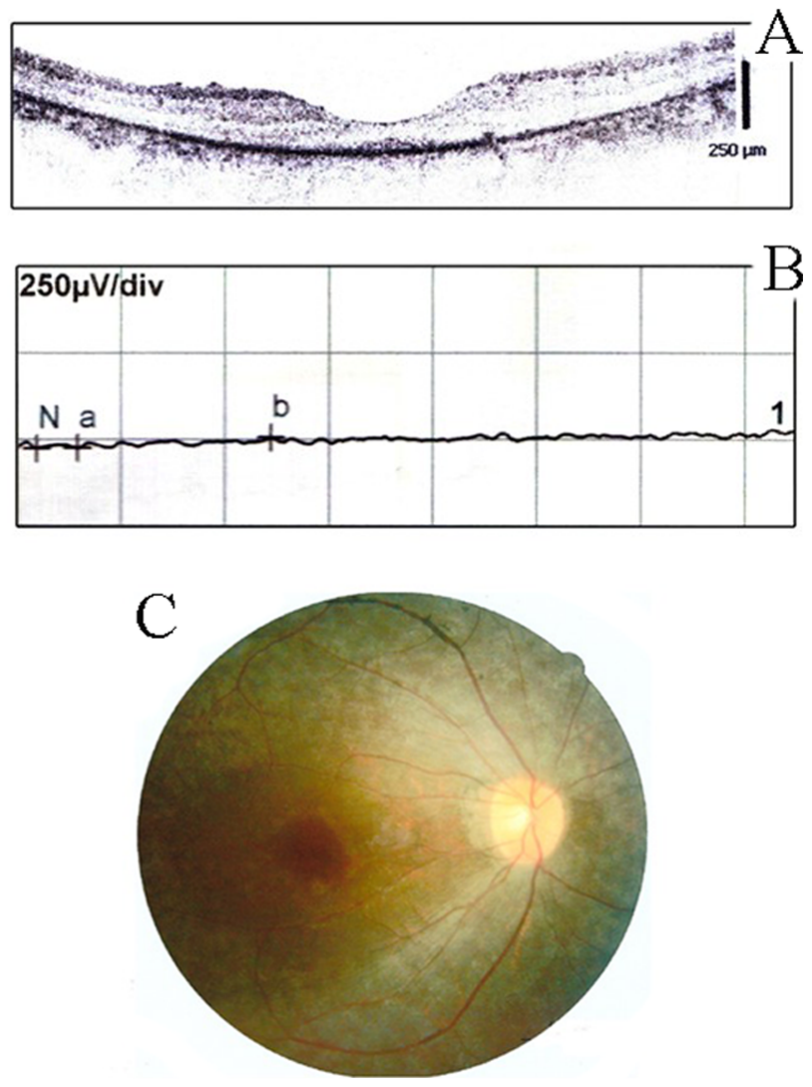
Clinical examination. A. OCT examination demonstrated thinned retina in the proband patient with Usher syndrome (F3-III-15); B. ERG testing showed extinguished rod response; C. representative fundography of the patient.

Typical RP signs in the fundi, including bone-spicule hyperpigmentation and attenuated arteries, were observed in all patients. ERG showed extinguished response of the rods and OCT clearly displayed thinner retinal thickness and disorganized inner or outer segment structure (Figure 2). Three proband patients complained progressive night blindness and mild deafness, indicating a diagnosis of type II Usher syndrome. However, another two probands clearly showed a vertigo symptom, supporting a type III diagnosis (Table 1). Among these patients, more severe visual dysfunction was present in the elders. These clinical evidences suggested diagnosis of Usher syndrome type II or III.

**Table 1:** Clinical manifestations of the proband patients.

Proband	M/F	Age	Onset	NB	Deaf	Vertigo	BCVA (R/L)
F1-III-2	M	29	5	+	+	−	0.7/0.8
F2-III-2	F	33	<5	+	+	−	0.3/0.3
F3-III-15	F	31	10	+	+	+	0.15/0.15
F4-III-4	F	40	<5	+	+	−	0.2/0.3
F5-II-4	F	65	<5	+	+	+	FC/0.1*

M, male; F, female; NB, night blindness; BCVA, best corrected visual acuity; R, right eye, L, left eye. Asterisk indicates BCVA after cataract surgery in the left eye.

### Targeted Exome Sequencing Identified Candidate Mutations

We performed targeted exome resequencing of 144 genes implicated in inherited retinal degeneration. For the samples subjected to TES, the average sequencing depths on the targeted regions were yielded from 104.38 to 140.13 (Table S4). Each sample had more than 98.0% targeted regions covered. Meanwhile, coverage of targeted exons for >10 reads were ranged from 90.7% to 92.3% and >20 reads from 81.6% to 85.0%. By using the SOAPsnp program [8], an average of 474 to 650 variants was identified for each sample. Among them, non-synonymous variants were ranged from146 to 178 including missense, nonsense and splicing variants. The amounts were further narrowed down to 15–27 respectively through excluding the variants reported in the HapMap 28 and the SNP release of the 1000 Genome Project with a MAF>0.05. In case of the missense variants, computational prediction by four algorithms (PolyPhen, SIFT, PANTHER and Pmut) and consistency of genetic transmission mode further confined the number of candidate mutations to less than 8. For the 6–27 coding InDels initially identified in the samples using the GATK program [11], we totally identified 2 variants based on the sample filtering strategy. By the stepwise approach as described, candidate mutations in all patients were successfully identified.

### Expanded Familial Validation and Sanger Sequencing Confirmation

Among the 5 USH families, pedigrees F1 and F3 (Figure 1) showed a pseudo-dominant transmission, which is definitely different with previous reports [2]. In family F1, both the proband (F1-III-2) and his father (F1-II-4) were diagnosed as typical USH based on their clinical features. Since the F1-I-1 and F1-I-2 were completely normal, it is thus conceivable that F1-II-4 carried a *de novo* dominant mutation, which was afterward transmitted to F1-III-2. Family F3 also showed a genetic continuity which was considered as a dominant pedigree. However, the TES results of the two probands were probably indicated to be the *USH2A* gene defects. To validate the TES results from proband, we extended the same procedure to the parents and a sibling in the F1 pedigree. Interestingly, expanded familial validation successfully revealed two compound heterozygous *USH2A* mutations (C3416G and R3484X) in the affected father (F1-II-4), a heterozygous *USH2A* mutation (Q3157X) in the unaffected mother (F1-II-5) and a heterozygous *USH2A* mutation (R3484X) in the unaffected sibling (F1-III-3), while the proband (F1-III-2) harbored two compound heterozygous *USH2A* mutations (Q3157X and R3484X) (Figure 1). Sanger sequencing of the coding region further confirmed that the mutation (R3484X) was transmitted from the paternal grandfather (F1-I-1) and the mutation (C3416G) was inherited from F1-I-2. The mutation (Q3157X) in the unaffected F1-II-5 was derived from maternal grandfather (F1-I-3). These results suggested the occurrence of compound heterozygosity in *USH2A* for USH. Taken together, the two causatives mutations in the proband (F1-III-2) were inherited from the paternal grandfather and maternal grandfather respectively. Furthermore, the affected father (F1-II-4) harbored another mutation in *USH2A* leading to the disease. Co-segregation analysis was performed to confirm the extracted mutations in the other 4 pedigrees. As a result, all mutations were confirmed to co-segregate well with the disease in these families. In the pseudo-dominant family F3, both the proband (F3-III-15) and the affected sister (F3-III-13) were revealed to have two compound heterozygous mutations, c.538T>C (p.S180P) and IVS48+1G>A. The splice site mutation was transmitted from proband to her unaffected son (F3-IV-2). It was suspected that the deceased affected mother (F3-II-6) carried another unknown mutation in the *USH2A* gene. In family F2, the proband (F2-III-2) was found to carry three mutations. This rare condition was further confirmed by intra-familial validation. Two heterozygous mutations, c.15427C>T (p.R5143C) and c.5581G>T (p.G1861S), were confirmed in the elder daughter (F2-IV-1) who was unaffected, which indicates the bi-mutation (c.5581G>A and c.15427C>T) in the maternal allele; while another frameshift mutation, c.8602delA, was identified in the unaffected younger daughter (F2-IV-2). In the pedigree F4, two affected siblings (F4-III-2 and F4-III-4) were revealed with the same heterozygous mutations, c.8212G>A (p.D2738N) and c.5528C>T (p.P1843L). In the last pedigree F5, a novel homozygous frameshift mutation (c.4383delT) was identified in the proband (F5-II-4). The unaffected son (F5-III-3) was proved to be a carrier. Taken together, causative mutations (Tables 2, S5 and Figure S1) were successfully finalized in all USH families via expanded TES, Sanger sequencing, and co-segregation analysis.

**Table 2:** Identified mutations in *USH2A* gene.

Family	Proband	Phen otype	Mutation	Type	Amino acid	Reported
F1	III-2	+	c.10450C>T	hetero	R3484X	Reported
			c.9469C>T	hetero	Q3157X	Reported
F2	III-2	+	c.5581G>A	hetero	G1861S	Novel
			c.15427C>T	hetero	R5143C	Novel
			c.8602delA	hetero	frameshift	Novel
F3	III-15	+	c.538T>T	hetero	S180P	Reported
			IVS48+1G>A	hetero	Splice site	Novel
F4	III-4	+	c.8212G>A	hetero	D2738N	Novel
			c.5528C>T	hetero	P1843L	Novel
F5	II-4	+	c.4383delT	homo	frameshift	Novel

In this study a total of 11 identified mutations were presumed to be pathological. Both the nonsense and frameshift mutations were predicted to create a premature stop codon, strongly indicating the protein dysfunction. The splicing site mutation, IVS48+1G>A, is predicted to be damaging in silico. In addition, all missense mutations were located in highly conserved (Figure S2) and functional domains as reported previously [14]. Taken together, these results strongly indicated disease-causing mutations in the *USH2A* gene.

### Mutation Spectrum in the *USH2A* Gene

**Figure 3:**
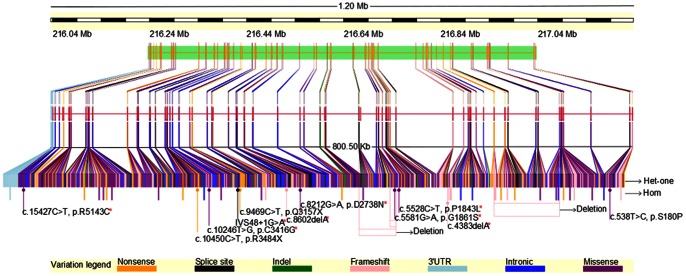
Mutation spectrum in the ***USH2A*** **gene.** Gene level overview of the summarized *USH2A* mutations. The gene is comprised of 71 exons which harbored 1000 distinct mutations so far have been reported. The new identified mutations are marked as arrows in the schema. Each color of the box in the figure represents a mutation type: orange, nonsense mutation; black, splice site mutation; green, indel; pink, frameshift mutation; sky blue, mutation in 3′UTR; blue, intronic mutation; purple, missense mutation. Red asterisk indicates novel mutation discovered in this study. Het-one, single heterozygous mutation; homo, homozygous mutation.

The mutation distribution had been summarized in previously reported *USH2A* mutations [15]–[17] and novel mutations identified in this study (Table 2). It is observed that the mutations spread over the whole region of the gene. Mutations identified in the present study, including the new ones, did not show specific distribution, suggesting that it may be absence of mutation hot spot in the *USH2A* in the population (Figure 3).

## Discussion

Usher syndrome is a severe disease with significant vision and hearing impairments. The prevalence worldwide ranged from 1/12,500 to 1/29,000 as previous studies have been shown [1]. Based on the phenotypic characterization, the disease has been classified into three subtypes. Seven loci (USH1B-H) have been mapped and five genes have been identified for type 1, four loci (USH2A-D) have been mapped and three genes have been identified for type 2, and two genes (USH3A, USH3B) have been identified for type 3. However, a number of studies have demonstrated phenotypic variation and crossover of the phenotype-genotype subtyping [18]. Together with the fact that a large number of coding exons exist in these genes, traditional screening of each region is infeasible for clinical application. In this study, we thus attempted to target on a group of genes responsible for inherited retinal degeneration, including RP and USH. As a result, we have proved deep exome sequencing of target 144 known causative genes of inherited retinal degeneration can serve as a fast and efficient way to perform the diagnosis. The cost was much lower compared with direct sequencing in a conservative estimation, while the amount of work is saved even more. Notably, only a single sample from the proband in each family is sufficient to identify causative mutation and intra-familiar mutation validation and co-segregation analysis can enhance the finalization.

In our USH families, mutations in the *USH2A* gene predispose the disease, suggesting the high prevalence of the *USH2A* mutation in Chinese USH population. Meantime, unaffected individuals, such as F1-II-5 from a completely normal family, were eventually found to carry a heterozygous mutation. It is worthwhile to be underlined that eight mutations have not yet been reported so far among the 11 mutations identified in this study. As there are few reports of molecular diagnosis on Chinese USH families [19], our results indicate a distinctive mutation spectrum in the population, which may require further investigation in more cohorts.

It is notable that in our strategy revealed genetic mutations in several extraordinary cases in this study. USH is well known as an autosomal recessive disease, however, among our families, pedigree F1 showed a successive transmission in a pseudo-dominant trait. This unusual case raised a question whether the patient F1-II-4 carried a *de novo* dominant mutation. Our results well addressed an autosomal recessive trait based on the discovery of a heterozygous mutation which is easily ignored in the proband's mother (F1-II-5). In family F2, we identified three distinct *USH2A* mutations in the proband (F2-III-1). In these cases, targeted exome sequencing allows excavating complete information of genetic defects which may be undetectable by traditional methods.

In the present study, we have successfully performed genetic diagnosis of Usher syndrome by utilization of NGS and have proved that it can serve as a rapid, high-throughput and efficient screening strategy. In brief, targeted exome sequencing of the 144 known genes is sufficient and clinically utilizable to comprehensively reveal genetic defects in patients with genetic retinal disease.

## Supporting Information

Figure S1 Identified mutations confirmed by Sanger sequencing. Corresponding chromatograms showing mutant and wild-type alleles are as indicated.
(TIF)

Figure S2 Conserved amino acid sequence. Conservation of amino acid residue across species is highlighted.
(TIF)

Table S1 Known causative genes responsible for Usher syndrome.
(DOC)

Table S2 List of the genes captured in the present study.
(DOC)

Table S3 List of all probes used to enrich for the target genes.
(XLSX)

Table S4 Data summary of the targeted exome resequencing. Asterisk indicates proband patient.
(DOC)

Table S5 Expanded familial validation.
(DOC)
