# Supplementary figures and images for: Targeted Exome Sequencing Identified Novel USH2A Mutations in Usher Syndrome Families
Source: PLoS One. 2013 May 30;8(5):e63832. doi: 10.1371/journal.pone.0063832 (PMC3667821; doi:10.1371/journal.pone.0063832)

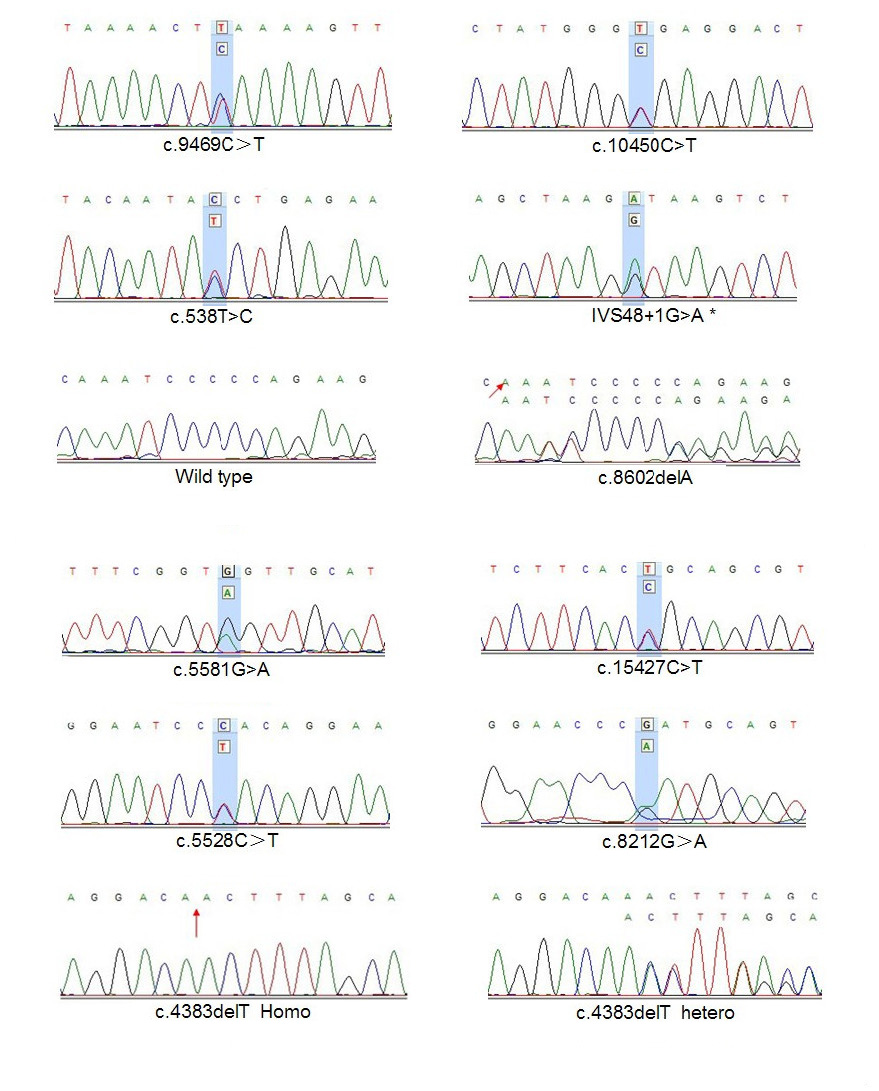

Supplement: Figure S1 — Identified mutations confirmed by Sanger sequencing. Corresponding chromatograms showing mutant and wild-type alleles are as indicated. (TIF) [file pone.0063832.s001.tif]

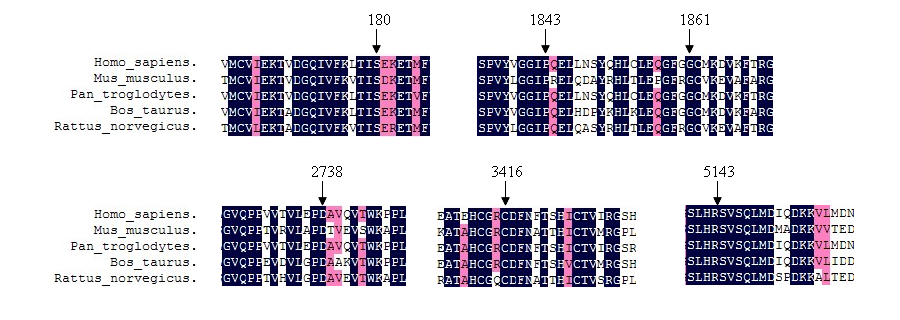

Supplement: Figure S2 — Conserved amino acid sequence. Conservation of amino acid residue across species is highlighted. (TIF) [file pone.0063832.s002.tif]
