# Supplementary material for: Targeted Exome Sequencing Identified Novel USH2A Mutations in Usher Syndrome Families
Source: PLoS One. 2013 May 30;8(5):e63832. doi: 10.1371/journal.pone.0063832 (PMC3667821; doi:10.1371/journal.pone.0063832)
Supplement: Table S1 — Known causative genes responsible for Usher syndrome. (DOC) [file pone.0063832.s003.doc]

**Supplemental table 1. Known causative genes responsible for Usher syndrome.**

| **No.** | **Gene** | **Location** | **Exon No.** | **Length (bp)** | **Amino acid** |
| --- | --- | --- | --- | --- | --- |
| 1 | MYO7A | 11q13.5 | 48 | 6648 | 2215 |
| 2 | USH1C | 11p14.3 | 27 | 2700 | 899 |
| 3 | CDH23 | 10q22.3 | 22 | 3345 | 1114 |
| 4 | PCDH15 | 10q21.1 | 32 | 5868 | 1955 |
| 5 | USH1G | 17q25.1 | 3 | 1386 | 461 |
| 6 | USH2A | 1q41 | 71 | 15609 | 5202 |
| 7 | GPR98 | 5q13 | 90 | 18921 | 6306 |
| 8 | DFNB31 | 9q32 | 12 | 2724 | 907 |
| 9 | CLRN1 | 3q25 | 3 | 699 | 232 |
| 10 | HARS | 5q31.3 | 13 | 1530 | 509 |
| **Total** | | | **321** | **59430** | **19800** |
