# Supplementary material for: Targeted Exome Sequencing Identified Novel USH2A Mutations in Usher Syndrome Families
Source: PLoS One. 2013 May 30;8(5):e63832. doi: 10.1371/journal.pone.0063832 (PMC3667821; doi:10.1371/journal.pone.0063832)
Supplement: Table S2 — List of the genes captured in the present study. (DOC) [file pone.0063832.s004.doc]

**Supplemental table 2. List of the genes captured in the present study.**

| **No.** | **Gene symbole** | **Chromosome** | **Coding size (bp)** | **Exon No.** |
| --- | --- | --- | --- | --- |
| 1 | ABCA4 | 1 | 6822 | 48 |
| 2 | ABCC6 | 16 | 4512 | 31 |
| 3 | ADAM9 | 8 | 2460 | 22 |
| 4 | AHI1 | 6 | 3591 | 26 |
| 5 | AIPL1 | 17 | 1155 | 6 |
| 6 | ALMS1 | 2 | 12504 | 23 |
| 7 | ARL6 | 3 | 561 | 7 |
| 8 | ATXN7 | 3 | 2838 | 12 |
| 9 | BBS1 | 11 | 1782 | 17 |
| 10 | BBS10 | 12 | 2172 | 2 |
| 11 | BBS12 | 4 | 2133 | 1 |
| 12 | BBS2 | 16 | 2166 | 17 |
| 13 | BBS4 | 15 | 1560 | 16 |
| 14 | BBS5 | 2 | 1026 | 12 |
| 15 | BBS9 | 7 | 2559 | 20 |
| 16 | BEST1 | 11 | 1815 | 8 |
| 17 | C1QTNF5 | 11 | 732 | 2 |
| 18 | C2ORF71 | 2 | 3867 | 2 |
| 19 | CA4 | 17 | 939 | 8 |
| 20 | CABP4 | 11 | 828 | 6 |
| 21 | CACNA1F | X | 5933 | 48 |
| 22 | CACNA2D4 | 12 | 3414 | 38 |
| 23 | CC2D2A | 4 | 4863 | 36 |
| 24 | CDH23 | 10 | 3345 | 22 |
| 25 | CDHR1 | 10 | 2580 | 17 |
| 26 | CEP290 | 12 | 7440 | 53 |
| 27 | CERKL | 2 | 1545 | 13 |
| 28 | CFH | 1 | 3696 | 22 |
| 29 | CLN3 | 16 | 1317 | 15 |
| 30 | CLRN1 | 3 | 738 | 4 |
| 31 | CNGA1 | 4 | 2280 | 9 |
| 32 | CNGB1 | 16 | 900 | 12 |
| 33 | CNGB3 | 8 | 2430 | 18 |
| 34 | COL11A1 | 1 | 5421 | 67 |
| 35 | COL2A1 | 12 | 4464 | 54 |
| 36 | COL9A1 | 6 | 2766 | 38 |
| 37 | CRB1 | 1 | 3885 | 10 |
| 38 | CRX | 19 | 900 | 3 |
| 39 | DFNB31 | 9 | 2724 | 12 |
| 40 | DHDDS | 1 | 1002 | 8 |
| 41 | PDZD7 | 10 | 1554 | 9 |
| 42 | EFEMP1 | 2 | 1482 | 10 |
| 43 | ELOVL4 | 6 | 945 | 6 |
| 44 | EYS | 6 | 1860 | 9 |
| 45 | FAM161A | 2 | 2151 | 7 |
| 46 | FLVCR1 | 1 | 1668 | 10 |
| 47 | FSCN2 | 17 | 1479 | 5 |
| 48 | GNAT1 | 3 | 1053 | 8 |
| 49 | GPR98 | 5 | 18921 | 90 |
| 50 | GRK1 | 13 | 2101 | 7 |
| 51 | GRM6 | 5 | 2634 | 10 |
| 52 | GUCA1A | 6 | 606 | 4 |
| 53 | GUCA1B | 6 | 603 | 4 |
| 54 | GUCY2D | 17 | 3312 | 18 |
| 55 | HMCN1 | 1 | 16908 | 107 |
| 56 | IDH3B | 20 | 702 | 6 |
| 57 | IMPDH1 | 7 | 1530 | 14 |
| 58 | IMPG2 | 3 | 3726 | 15 |
| 59 | INPP5E | 9 | 1935 | 10 |
| 60 | INVS | 9 | 3198 | 16 |
| 61 | IQCB1 | 3 | 1797 | 13 |
| 62 | JAG1 | 20 | 3657 | 26 |
| 63 | KCNJ13 | 2 | 285 | 2 |
| 64 | KCNV2 | 9 | 1638 | 2 |
| 65 | KLHL7 | 7 | 501 | 5 |
| 66 | LCA5 | 6 | 2094 | 7 |
| 67 | LRAT | 4 | 693 | 2 |
| 68 | LRP5 | 11 | 4848 | 23 |
| 69 | MAK | 6 | 1872 | 13 |
| 70 | MERTK | 2 | 3000 | 19 |
| 71 | MKKS | 20 | 1713 | 4 |
| 72 | MKS1 | 17 | 1680 | 18 |
| 73 | MTTP | 4 | 2685 | 18 |
| 74 | MYO7A | 11 | 6648 | 48 |
| 75 | NPHP1 | 2 | 2202 | 20 |
| 76 | NPHP3 | 3 | 3993 | 27 |
| 77 | NPHP4 | 1 | 4281 | 29 |
| 78 | NR2E3 | 15 | 2102 | 8 |
| 79 | NRL | 14 | 741 | 2 |
| 80 | NYX | X | 1446 | 2 |
| 81 | OFD1 | X | 3039 | 23 |
| 82 | OPA1 | 3 | 2883 | 28 |
| 83 | OPA3 | 19 | 543 | 2 |
| 84 | OTX2 | 14 | 894 | 3 |
| 85 | PANK2 | 20 | 1713 | 7 |
| 86 | PAX2 | 10 | 1254 | 11 |
| 87 | PCDH15 | 10 | 5874 | 33 |
| 88 | PDE6A | 5 | 2583 | 22 |
| 89 | PDE6B | 4 | 2562 | 22 |
| 90 | PDE6C | 10 | 2577 | 22 |
| 91 | PDE6G | 17 | 264 | 3 |
| 92 | PEX1 | 7 | 3852 | 24 |
| 93 | PEX7 | 6 | 972 | 10 |
| 94 | PHYH | 10 | 1017 | 9 |
| 95 | PITPNM3 | 17 | 2925 | 20 |
| 96 | PRCD | 17 | 165 | 3 |
| 97 | PROM1 | 4 | 2529 | 25 |
| 98 | PRPF3 | 1 | 2052 | 15 |
| 99 | PRPF31 | 19 | 1500 | 13 |
| 100 | PRPF6 | 20 | 2826 | 21 |
| 101 | PRPF8 | 17 | 7008 | 42 |
| 102 | PRPH2 | 6 | 1041 | 3 |
| 103 | PXMP3 | 8 | 918 | 1 |
| 104 | RAX2 | 19 | 555 | 2 |
| 105 | RBP3 | 10 | 3744 | 4 |
| 106 | RD3 | 1 | 588 | 2 |
| 107 | RDH12 | 14 | 951 | 7 |
| 108 | RDH5 | 12 | 957 | 4 |
| 109 | RGR | 10 | 762 | 6 |
| 110 | RHO | 3 | 1047 | 5 |
| 111 | RIMS1 | 6 | 5079 | 34 |
| 112 | RLBP1 | 15 | 954 | 7 |
| 113 | ROM1 | 11 | 1056 | 3 |
| 114 | RP1 | 8 | 6471 | 3 |
| 115 | RP1L1 | 8 | 7203 | 3 |
| 116 | RP2 | X | 1053 | 5 |
| 117 | RP9 | 7 | 666 | 6 |
| 118 | RPE65 | 1 | 1602 | 14 |
| 119 | RPGR | X | 3459 | 15 |
| 120 | RPGRIP1 | 14 | 3861 | 24 |
| 121 | RPGRIP1L | 16 | 3948 | 26 |
| 122 | SAG | 2 | 1218 | 15 |
| 123 | SDCCAG8 | 1 | 2142 | 18 |
| 124 | SEMA4A | 1 | 1890 | 12 |
| 125 | SLC24A1 | 15 | 3300 | 9 |
| 126 | SNRNP200 | 2 | 6411 | 45 |
| 127 | SPATA7 | 14 | 1704 | 11 |
| 128 | TIMM8A | X | 294 | 2 |
| 129 | TIMP3 | 22 | 636 | 5 |
| 130 | TMEM126A | 11 | 588 | 4 |
| 131 | TOPORS | 9 | 2943 | 2 |
| 132 | TREX1 | 3 | 1110 | 1 |
| 133 | TRIM32 | 9 | 1962 | 1 |
| 134 | TRPM1 | 15 | 4812 | 26 |
| 135 | TTC8 | 14 | 1548 | 15 |
| 136 | TTPA | 8 | 837 | 5 |
| 137 | TULP1 | 6 | 1629 | 15 |
| 138 | UNC119 | 17 | 723 | 5 |
| 139 | USH1C | 11 | 2700 | 27 |
| 140 | USH1G | 17 | 1386 | 2 |
| 141 | USH2A | 1 | 15609 | 71 |
| 142 | VCAN | 5 | 10191 | 14 |
| 143 | WFS1 | 4 | 2673 | 7 |
| 144 | ZNF513 | 2 | 1440 | 3 |
| **Total** | | | **394787** | **2285** |
