# Supplementary material for: Targeted Exome Sequencing Identified Novel USH2A Mutations in Usher Syndrome Families
Source: PLoS One. 2013 May 30;8(5):e63832. doi: 10.1371/journal.pone.0063832 (PMC3667821; doi:10.1371/journal.pone.0063832)
Supplement: Table S4 — Data summary of the targeted exome resequencing. Asterisk indicates proband patient. (DOC) [file pone.0063832.s006.doc]

**Supplemental table 4**. Quality of the targeted exome resequencing.

| **Sample** | **Aligned (%)** | **Fraction of effective bases on target** | **Average sequencing depth on target** | **Fraction of target covered with at least 1X** | **Fraction of target covered with at least 4X** | **Fraction of target covered with at least 10X** | **Fraction of target covered with at least 20X** |
| --- | --- | --- | --- | --- | --- | --- | --- |
| F1-III-2* | 94.37% | 31.10% | 140.13 | 98.30% | 96.00% | 91.00% | 82.80% |
| F1-II-4 | 93.98% | 39.80% | 122.75 | 99.00% | 97.30% | 93.20% | 85.00% |
| F1-II-5 | 94.21% | 40.30% | 112.08 | 98.20% | 94.10% | 92.30% | 81.60% |
| F1-III-3 | 93.33% | 37.20% | 104.38 | 98.50% | 95.80% | 91.60% | 83.40% |
| F2-III-2* | 94.43% | 34.90% | 152.33 | 98.20% | 97.80% | 92.30% | 83.00% |
| F3-III-15* | 94.06% | 40.30% | 130.53 | 98.30% | 95.80% | 91.50% | 84.90% |
| F4-III-4* | 94.94% | 35.50% | 118.24 | 98.00% | 95.30% | 90.70% | 84.70% |
| F5-II-4* | 94.32% | 35.40% | 131.27 | 98.50% | 96.30% | 91.40% | 83.30% |
