# Supplementary material for: Targeted Exome Sequencing Identified Novel USH2A Mutations in Usher Syndrome Families
Source: PLoS One. 2013 May 30;8(5):e63832. doi: 10.1371/journal.pone.0063832 (PMC3667821; doi:10.1371/journal.pone.0063832)
Supplement: Table S5 — Expanded familial validation. (DOC) [file pone.0063832.s007.doc]

**Supplemental table 5**. Expanded familial validation.

| **Family** | **Subject** | **Disease** | **Mutation** | **Type** | **Amino acid** | **Reported** |
| --- | --- | --- | --- | --- | --- | --- |
| F1 | I-1 | - | c.10450C>T | hetero | R3484X | Reported |
|  | I-2 | - | c.10246T>G | hetero | C3416G | Novel |
|  | I-3 | - | c.9469C>T | hetero | Q3157X | Reported |
|  | I-4 | - | c.9469C>T | hetero | Q3157X | Reported |
|  | II-3 | - | c.10450C>T | hetero | R3484X | Reported |
|  | II-4 | + | c.10450C>T | hetero | R3484X | Reported |
|  |  |  | c.10246T>G | hetero | C3416G | Novel |
|  | II-5 | - | c.9469C>T | hetero | Q3157X | Reported |
|  | III-2 | + | c.10450C>T | hetero | R3484X | Reported |
|  |  |  | c.9469C>T | hetero | Q3157X | Reported |
|  | III-3 | - | c.10450C>T | hetero | R3484X | Reported |
| F2 | III-2 | + | c.5581G>A | hetero | G1861S | Novel |
|  |  |  | c.15427C>T | hetero | R5143C | Novel |
|  |  |  | c.8602delA | hetero | frameshift | Novel |
|  | III-4 | - | None |  |  |  |
|  | IV-1 | - | c.5581G>A | hetero | G1861S | Novel |
|  |  |  | c.15427C>T | hetero | R5143C | Novel |
|  | IV-2 | - | c.8602delA | hetero | frameshift | Novel |
| F3 | III -11 | - | None |  |  |  |
|  | III -13 | + | c.538T>C | hetero | S180P | Reported |
|  |  |  | IVS48+1G>A | hetero | Splice site | Novel |
|  | III -15 | + | c.538T>C | hetero | S180P | Reported |
|  |  |  | IVS48+1G>A | hetero | Splice site | Novel |
|  | III -16 | - | None |  |  |  |
|  | IV -2 | - | IVS48+1G>A | hetero | Splice site | Novel |
| F4 | III-2 | + | c.8212G＞A | hetero | D2738N | Novel |
|  |  |  | c.5528C＞T | hetero | P1843L | Novel |
|  | III-4 | + | c.8212G＞A | hetero | D2738N | Novel |
|  |  |  | c.5528C＞T | hetero | P1843L | Novel |
| F5 | II-4 | + | c.4383delT | homo | frameshift | Novel |
|  | III-3 | - | c.4383delT | hetero | frameshift | Novel |
